# Supplementary figures and images for: Genetic association of serum lipids and lipid-modifying targets with endometriosis: Trans-ethnic Mendelian-randomization and mediation analysis
Source: PLoS One. 2024 May 31;19(5):e0301752. doi: 10.1371/journal.pone.0301752 (PMC11142702; doi:10.1371/journal.pone.0301752)

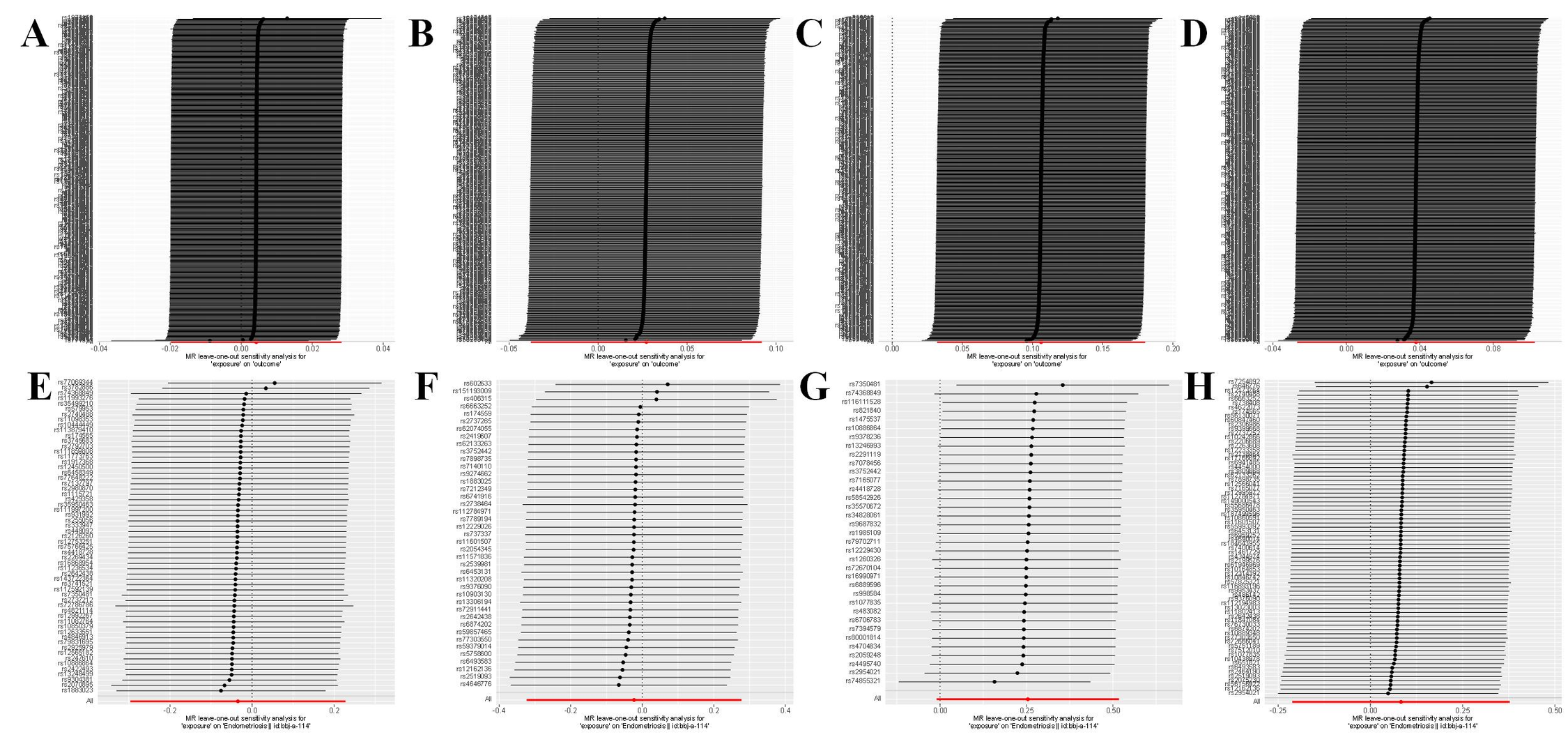

Supplement: S1 Fig — (A) HDL-C on EMS-EUR (B) LDL-C on EMS-EUR (C) TG on EMS-EUR (D) TC on EMS-EUR (E) HDL-C on EMS-EAS (F) LDL-C on EMS-EAS (G) TG on EMS-EAS (H) TC on EMS-EAS. LDL-C, Low-Density Lipoprotein Cholesterol; HDL-C, High Density Lipoprotein Cholesterol; TG, Triglyceride; TC, total cholesterol; EMS, endometriosis; EUR, European; EAS, East Asian. (TIF) [file pone.0301752.s006.tif]

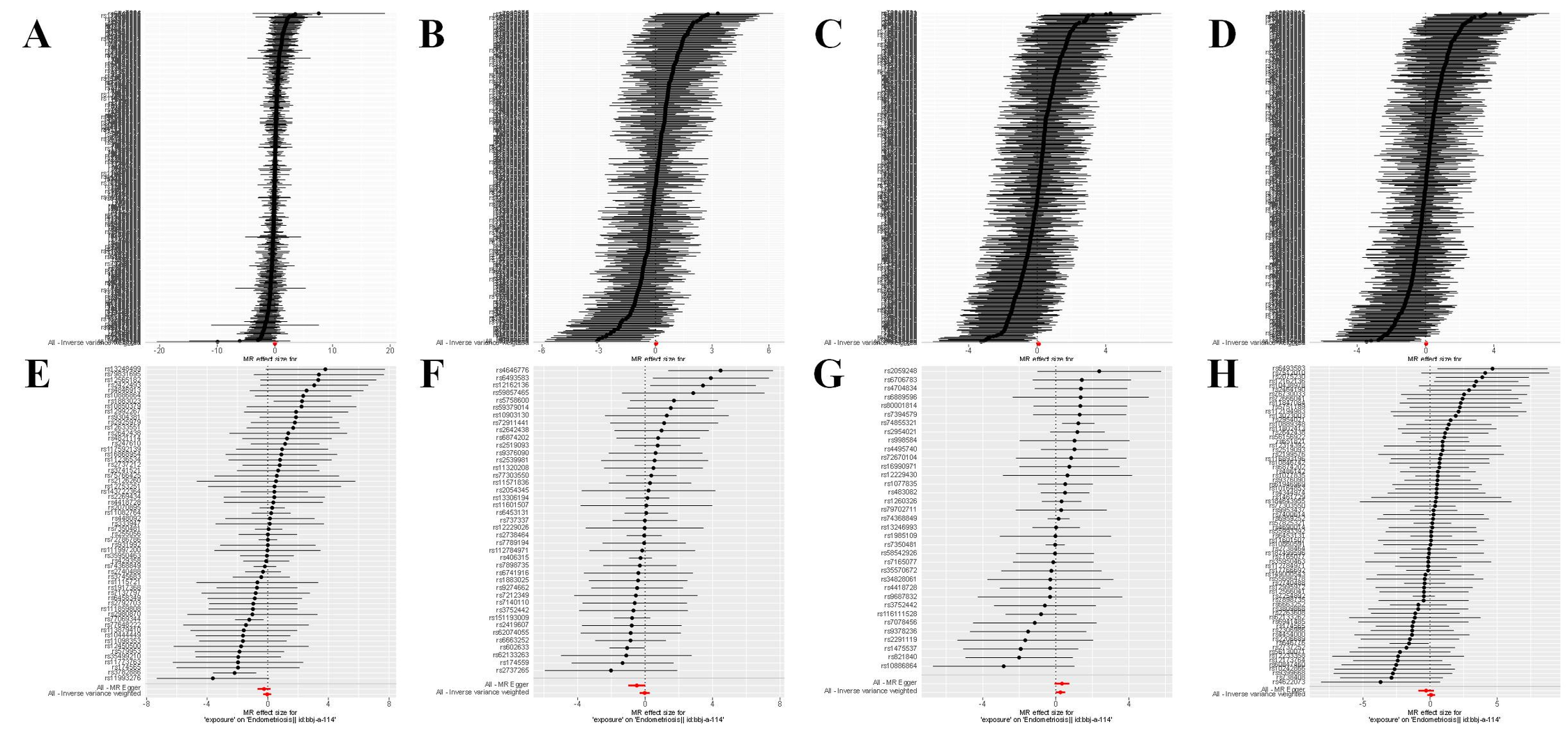

Supplement: S2 Fig — (A) HDL-C on EMS-EUR (B) LDL-C on EMS-EUR (C) TG on EMS-EUR (D) TC on EMS-EUR (E) HDL-C on EMS-EAS (F) LDL-C on EMS-EAS (G) TG on EMS-EAS (H) TC on EMS-EAS. LDL-C, Low-Density Lipoprotein Cholesterol; HDL-C, High Density Lipoprotein Cholesterol; TG, Triglyceride; TC, total cholesterol; EMS, endometriosis; EUR, European; EAS, East Asian. (TIF) [file pone.0301752.s007.tif]

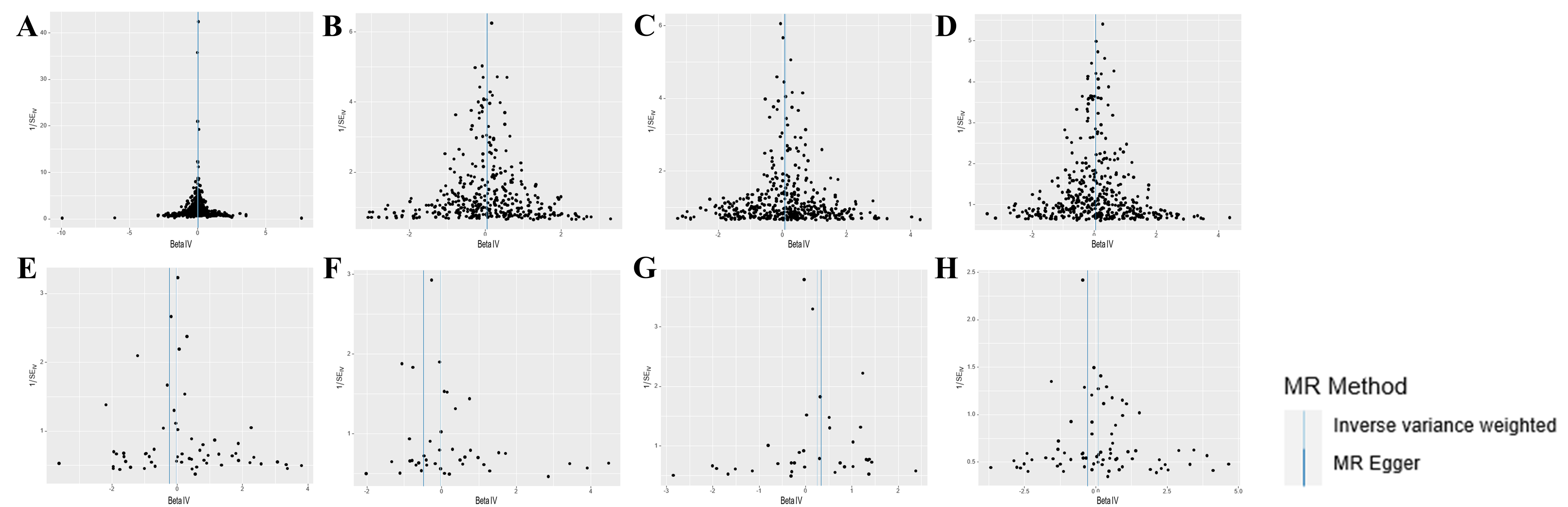

Supplement: S3 Fig — (A) HDL-C on EMS-EUR (B) LDL-C on EMS-EUR (C) TG on EMS-EUR (D) TC on EMS-EUR (E) HDL-C on EMS-EAS (F) LDL-C on EMS-EAS (G) TG on EMS-EAS (H) TC on EMS-EAS. LDL-C, Low-Density Lipoprotein Cholesterol; HDL-C, High Density Lipoprotein Cholesterol; TG, Triglyceride; TC, total cholesterol; EMS, endometriosis; EUR, European; EAS, East Asian. (TIF) [file pone.0301752.s008.tif]
