# Supplementary material for: Genetic association of serum lipids and lipid-modifying targets with endometriosis: Trans-ethnic Mendelian-randomization and mediation analysis
Source: PLoS One. 2024 May 31;19(5):e0301752. doi: 10.1371/journal.pone.0301752 (PMC11142702; doi:10.1371/journal.pone.0301752)
Supplement: Attachment — Submitted filename: Response to Reviewers.docx [file pone.0301752.s009.docx]

Dear Editors and Reviewers:

On behalf of all contributing authors, I would like to sincerely thank you for your letter and the constructive comments provided by the reviewers on our article titled " Genetic association of serum lipids and lipid-modifying targets with endometriosis: Trans-ethnic Mendelian-randomization and Mediation Analysis" (Manuscript ID: PONE-D-23-40604). We highly value these comments, as they have greatly contributed to the enhancement of our manuscript. Based on the suggestions from the associate editor and reviewers, we have made extensive revisions to our draft and added additional data to substantiate our findings. In this revised version, all changes to our manuscript are highlighted in yellow text. Following this letter, we provide point-by-point responses to the comments from the associate editor and the reviewer.

**Reviewer #1:**

Introduction
1.Line 105: “associated with positive lipid levels” – do you mean “positively associated with lipid levels?”

Response: Thank you for pointing out the ambiguity in Line 105 of our manuscript. In light of your feedback, we have revised the sentence for clarity. It now accurately reflects our intent to investigate blood lipid levels with a significant causal link to EMS and to identify lipid-lowering gene targets. This revision aims to more precisely articulate the study's objective of elucidating the causal relationship between these gene targets and EMS. We appreciate your valuable input in enhancing the clarity and precision of our manuscript.

Revised: The following sentence should be amended to read: "Further, it sought to identify lipid-lowering gene targets associated with positive lipid levels to explore their causal relationship with EMS. with EMS." to "Further, this study delved into blood lipid levels that have a significant causal link with EMS, identifying lipid-lowering gene targets. The objective was to clarify the causal nexus between lipid levels influenced by these gene targets and EMS. "

Materials and methods
1.Line 140 – What LD reference panel was used for variant selection for MR? i.e. for calculating LD to pick independent variants

Response: In our MR analysis, we utilized the TwoSampleMR package, which incorporates the 1000 Genomes Phase 3 reference panel for linkage disequilibrium (LD) calculations. This reference panel includes multiple super-populations, covering a broad spectrum of genetic diversity, including East Asian populations. The function within the package searches for specified SNPs in the chosen super-population and then creates an LD matrix of r values. We ensured the uniqueness of the selected SNPs by considering the LD structure specific to the relevant population, thus reducing biases linked to linkage disequilibrium. This approach allowed us to maintain the integrity of our analysis by using a comprehensive and globally representative genetic reference, ensuring that our SNP selection was robust and appropriate for our MR study

2.Line 141 – please provide a citation for the F statistic formula.

Response: Thank you for pointing out the oversight regarding the citation for the F statistic formula. We acknowledge this lapse and have now included the appropriate reference in line 141.

3.Line 143 – “an F-statistic exceeding 10 was necessary to eliminate weak instruments” – this gives the impression you removed SNPs with F stat >10, which is not true based on your later statements that indicate you calculated average F statistics (e.g. line 277-278: “The average F statistics for these IVS all exceeded 90”. Please rephrase.

Response: Thank you for highlighting the inconsistency in our description regarding the F-statistic. We have now revised the text to more accurately reflect our methodology.

Revised: Amend "An F-statistic exceeding 10 was necessary to eliminate weak instruments, thereby verifying the robustness of the instruments" to "To ensure the robustness of our IVs, we excluded any SNPs with an F-statistic below 10, as values lower than this threshold are indicative of weak instruments."

4.Line 157-159 – What was the clumping window used for the target gene SNP clumping?

Response: Thank you for your query regarding the clumping window used for the target gene SNP clumping in our analysis. I appreciate your attention to detail and acknowledge the lack of clarity in our initial description. We have revised the manuscript to accurately reflect the methodology employed.

Revised: "To maximize the instrumental strength of each drug, SNPs utilized as instruments were allowed to exhibit low LD with each other (r2 < 0.30)". Added "localized within ±100 kb windows from gene region" after the paragraph.

5.Line 188 – do you mean GWAS summary statistics were sourced from the UKB? If so, in what publication were the summary statistics generated? Also, were the testosterone, E2 and SHBG data were female-specific?

Response: Thank you for your question regarding the source of GWAS summary statistics used in our analysis. Indeed, the data for testosterone, estradiol (E2), and sex hormone-binding globulin (SHBG) were female and sourced from the UK Biobank (UKB). The summary-level GWAS data for these biomarkers can be accessed from the following links: Total Testosterone (https://gwas.mrcieu.ac.uk/datasets/ieu-b-4864/), E2 (https://gwas.mrcieu.ac.uk/datasets/ieu-b-4872/), and SHBG (https://gwas.mrcieu.ac.uk/datasets/ieu-b-4870/). Each dataset is explicitly noted as 'Output from UK Biobank - stratified by sex', ensuring the specificity of the data to female participants.

6.Line 200 – “while MR-Egger is predicated on the assumption of total invalidity of these variants[28].” This could be better explained as MR-Egger allows for a directional pleiotropic effect on the outcome.

Response: Thank you for your valuable feedback on our description of the MR-Egger method. I agree that the original phrasing might have oversimplified the method's premise. We have revised the text to more accurately reflect that MR-Egger allows for the detection and correction of directional pleiotropic effects from the instrumental variables on the outcome.

Revised: Replace "while MR-Egger is predicated on the assumption of total invalidity of these variants" with "While MR-Egger method allows for the identification and adjustment of directional pleiotropic effects, assuming that the instrument-outcome relationship is independent of the exposure".

7.Line 230-232: was 0.7 or 0.8 the threshold?

Response: Thank you for pointing out my problem, which has now been changed from 0.7 to 0.8 with additional citations

8.Line 247 – what are the two types of horizontal pleiotropy you are referring to?
Response: "Thank you for your question regarding the types of horizontal pleiotropy mentioned in line 247. The two types of horizontal pleiotropy we are referring to are detected through traditional sensitivity analyses using MR-Egger and MR-PRESSO methods. Specifically, MR-Egger identifies horizontal pleiotropy when its intercept significantly deviates from zero (*P* < 0.05), suggesting that some instrumental variables might exert effects on the outcome that are independent of the exposure. On the other hand, MR-PRESSO detects horizontal pleiotropy through a global test (Global *P* < 0.05), identifying outliers that could be indicative of pleiotropic effects. In cases where horizontal pleiotropy is detected, we further employ CAUSE analysis to strengthen the positive findings while mitigating the potential for false positives due to pleiotropic effects. This approach ensures that our results are robust and not unduly influenced by horizontal pleiotropy."

Results
1.Line – 269 – “Outliers detected by MR-PRESSO were excluded from the final analysis” – what final analysis? Were the outliers detected by MR-PRESSO also removed from the other methods (e.g. IVW, MRE etc.)? Or do you mean the MR-PRESSO result is the outlier-adjusted result? Please clarify.

Response: Thank you for seeking clarification regarding the use of MR-PRESSO in our analysis. By 'final analysis', we refer to the analyses conducted after the iterative process of IV selection, where outliers detected by MR-PRESSO were excluded. To address your specific query, yes, the outliers identified by MR-PRESSO were indeed removed from all subsequent analyses, including those using other methods like IVW and MR-Egger. This means that the results presented for these methods in the sensitivity analysis are based on the refined set of IVs, post-outlier removal by MR-PRESSO. Essentially, the MR-PRESSO’s outlier-adjusted result reflects this exclusion of outliers, ensuring that the instrumental variables used in our final analysis are more robust and less likely to be influenced by pleiotropic effects.

Revised: Amend "Outliers detected by MR-PRESSO were excluded from the final analysis" to "Outliers identified by MR-PRESSO were excluded, and the subsequent analyses, were conducted with this refined set of IVs.

2.Line 271 – the process of calculating the F statistic have not reduced bias. Please reword.

Response: Thank you for pointing out the need for clarification regarding the interpretation of the F statistics in our manuscript. I appreciate your valuable feedback. We have now revised the statement to more accurately reflect the significance of the F statistics in our analysis.

Revised: Amend "The average F statistics for the IVs ranged from 121 to 372, indicating a significant reduction in bias due to weak instrumental variables. " to "The average F statistics for our IVs, ranging from 121 to 372, demonstrate their robustness, thus reducing the potential bias from weak instruments."

3.Figure 2 – Change axis labels on plots to indicate the specific exposure/outcome

Response: Thank you for your suggestion to improve the clarity of Figure 2. We have now updated the figure to include specific axis labels that clearly indicate the exposures and outcomes being analyzed. The revised Figure 2 has been re-uploaded to accurately reflect these changes and provide a clearer visual representation of our results.

4.Line 291- the confidence interval does not include the estimate

Response: Thank you for pointing out the discrepancy in the confidence interval reported in Line 291. Upon reviewing the data, I realized there was an error in the reported values. The correct confidence interval should be [1.033–1.198], which includes the estimate. I have amended this in the manuscript to reflect the accurate values and ensure the consistency of our statistical reporting. I appreciate your attention to detail in this matter.

Revised: Amend "Confidence Interval [CI] 1.033–1.098 " to "Confidence Interval [CI] 1.033–1.198 "

5.Line 310 – What supplementary methods? Where can these results be found?

Response: Thank you for your inquiry regarding the supplementary methods mentioned in Line 310. I apologize for any confusion caused. The results of all supplementary methods are comprehensively detailed in S3 Table of the supplementary material. This has been explicitly referenced at the end of the manuscript for ease of access. I appreciate your attention to this detail and hope that this clarification helps in locating the relevant results.

6.Line 311 – “negative causal associations” – this could be interpreted as a preventative effect, not a null effect. Please reword.

Response: Thank you for pointing out the potential misinterpretation in the phrasing 'negative causal associations' in Line 311. I realize that this could be misconstrued as indicating a preventative effect, which was not the intended meaning. I have now rephrased this to 'no significant causal associations' to accurately reflect the results where P-values and PFDR values were greater than 0.05, indicating a lack of statistically significant findings. This amendment should eliminate any confusion regarding the interpretation of the results. I appreciate your attention to clarity in our manuscript.

Revised: Amend " Further replication analyses and meta-analyses provided consistent evidence of negative causal associations " to " Further replication analyses and meta-analyses provided consistent evidence indicating no causal associations "

7.Line 322-324 – The “P > 0.05” I presume only applies to the first part of the sentence, not the second (“except for LDC-C…”). I suggest changing it to “MR-PRESSO and Cochran's Q statistics revealed no significant heterogeneity or horizontal pleiotropy in the results (P > 0.05), except for LDL-C, TC, and TG in EUR ancestry (P < 0.05).”
Response: Thank you for your suggestion to clarify the presentation of the results from MR-PRESSO and Cochran's Q statistics in Lines 322-324. I acknowledge the ambiguity in the original phrasing and have now revised this section as suggested. The revised sentence clearly differentiates the findings, highlighting the absence of significant heterogeneity or horizontal pleiotropy across most analyses (P > 0.05), while specifying the exceptions for LDL-C, TC, and TG in EUR ancestry (P < 0.05). This modification should provide a clearer and more accurate representation of our sensitivity analysis results. I appreciate your careful review and helpful feedback

Revised: Amend " MR-PRESSO and Cochran's Q statistics revealed no significant heterogeneity or horizontal pleiotropy in the results, except for LDL-C, TC, and TG in EUR ancestry (*P* > 0.05) " to " MR-PRESSO and Cochran's Q statistics revealed no significant heterogeneity or horizontal pleiotropy in the results (*P* > 0.05), except for LDL-C, TC, and TG in EUR ancestry (*P* < 0.05)."

8.Table 2 – are the estimates the effect of TG on endometriosis adjusted for the variable in the model column, or the variable in the Model column adjusted for TG? This could be made clearer. It would be useful to see the estimates of both exposures in the models.

Response: Thank you for your suggestion regarding the presentation of estimates for both exposures in the models. In our study, we focused on presenting the estimates of the primary exposure, TG, on EMS after adjusting for various confounders in the MVMR analysis. Based on our experience with previous MR studies[1–4], we found that focusing on the primary exposure allows for a more streamlined and clear presentation of results. Including estimates for each confounder's impact on EMS would significantly increase the complexity and length of the table, potentially obscuring the primary findings. Therefore, we have chosen to maintain the current format in the revised manuscript for clarity and conciseness. We hope this decision aligns with the overall aim of presenting clear and focused results, and we appreciate your understanding in this matter.

Revised: Original title "Table 2. Adjustment for MVMR analysis in TG on EMS (EUR)", modified title "Table 2. MVMR Analysis of TG on Endometriosis (EMS) in EUR Ancestry, Adjusted for Confounding Variables"

9.Line 323 – What statistic from MR-PRESSO indicated no heterogeneity and no horizontal pleiotropy?

Response: Thank you for your inquiry regarding the specific statistics from MR-PRESSO that indicated no significant heterogeneity and no horizontal pleiotropy in our results. The detailed values from MR-PRESSO and other sensitivity analyses are provided in S4 Table. I realize now that this reference might not have been clearly visible in the original text. To address this, I have made a revision to explicitly direct readers to S4 Table immediately after discussing the MR-PRESSO and Cochran's Q statistics results.

10.Line 326-327 – Does the CAUSE result tell you the result isn’t biased, or does it give you the causal effect with bias, if present, adjusted for? This is unclear from this sentence.

Response: Thank you for your question regarding the interpretation of the CAUSE analysis results in Line 326-327. I understand the need for clarification in this section. The purpose of the CAUSE analysis in our study was to assess whether the observed causal effect of TG on EMS in EUR ancestry was subject to potential biases, such as those arising from pleiotropy or heterogeneity. The CAUSE analysis results suggest that the positive findings we report are robust and not significantly influenced by such biases. I have revised the sentence to more clearly convey that the CAUSE analysis supports the robustness of our findings against potential biases. Your feedback has been invaluable in enhancing the clarity of our manuscript.

Revised: Amend＂CAUSE analysis of the causal model indicated that the causal effect of TG on EMS in EUR ancestry was not subject to bias＂to＂This analysis indicated that the observed causal effect of TG on EMS in EUR ancestry is robust and not likely to be biased by pleiotropy or heterogeneity＂

11.Line 332-335 – as you have listed these values in a table, it would read more coherently to not include them all in text.

Response: Thanks for pointing this out, it's all been removed now to increase the smoothness of the text reading

12.Supplementary Figure 3 – What is on the x axes? The label is unclear.
Response: Thank you for your question, have changed the label on the x-axis to Beta IV

Discussion
1.Line 367-369 – the causal effect of TG on EMS was found to be confounded by DBP, and mediated by SBP and TT. This means there is not strong evidence TG is causal of EMS, and suggests pleiotropic instruments may be present. The wording of this concluding sentence should be adjusted to reflect this.
Response: Thank you for your valuable feedback regarding the wording of our conclusions. Based on your suggestion, we have revised the concluding sentence to more accurately reflect the nuances of our findings. We now state that while our MR analysis indicates an association between higher levels of TG and increased EMS risk in EUR ancestry, this relationship is potentially confounded by DBP and mediated by factors such as SBP and TT. This revision aims to acknowledge the complexity of the causal relationship and the potential influence of confounding and mediating factors

Revised: Amend＂The MR findings corroborated previous epidemiological research, identifying a causal link between higher levels of TG and increased EMS risk in EUR ancestry, along with the mediating roles of SBP and TT＂to＂The MR indicates that higher levels of TG are associated with increased risk of EMS in individuals of EUR ancestry, but this relationship is confounded by DBP and mediated by factors such as SBP and TT＂

2.Can you explain the difference between the MVMR and mediation analysis results?
Response: Thank you for your question regarding the distinction between MVMR and Mediation MR analysis in our study. These two methods serve different purposes and offer distinct insights into the causal relationship between TG and EMS.

MVMR analysis aims to assess the independent effects of multiple exposures (or confounders) on an outcome. In our study, this approach evaluated the direct impact of the exposure (TG) on the EMS, while adjusting for several potential confounding variables. Our MVMR analysis revealed that after adjusting for DBP, the causal relationship between TG and EMS was no longer significant, suggesting that DBP may act as a confounder in the TG-EMS relationship. MVMR focuses on estimating the direct effect of a single exposure on an outcome, taking into account other potentially related exposures. This is crucial for identifying and controlling confounding factors, thereby enhancing our understanding of the relationship between a specific exposure and outcome[5]. On the other hand, Mediation MR analysis aims to explain how an exposure affects an outcome through a mediating variable. This type of analysis typically involves two main steps: estimating the impact of the exposure on the mediating variable using genetic instrumental variables, and then assessing the effect of the mediating variable on the outcome. Through these steps, mediation effects, or the indirect effects of the exposure on the outcome via the mediator, can be calculated. This method has the advantage of avoiding biases due to confounding factors while estimating different effects necessary for mediation analysis. In our case, the mediation analysis revealed the indirect impact of TG on EMS through mediators such as SBP and TT. Mediation MR focuses on understanding how an exposure indirectly influences an outcome through one or more mediators, revealing the biological or physiological pathways underlying the exposure-outcome relationship."

3.Need to discuss more limitations of analysis
- Sex-specific data?
- Age of participants in GWAS studies in comparison to age of onset of endometriosis?
- Power of east Asian sample?

Response: Thank you for highlighting the importance of discussing the limitations of our analysis more thoroughly. Following your suggestion, we have expanded the limitations section of our manuscript. We have now included a detailed discussion on the constraints related to the use of sex-combined GWAS data, which prevents sex-specific analyses. Additionally, we have addressed the limitation regarding the comparison of participant ages in the GWAS studies to the typical age of onset for endometriosis and acknowledged the potential impact this might have on our findings. We have also elaborated on the limited power of our analysis in the East Asian sample due to the smaller size of available GWAS data in this population. These amendments aim to provide a more comprehensive understanding of the potential limitations of our study and its implications. We appreciate your insightful comments which have significantly contributed to improving the quality of our manuscript.

Revised: Added "This is particularly relevant as our analysis could not differentiate the potentially unique causal relationships in males and females, nor could it account for the variations in disease risk and lipid levels across different ages. Finally, due to limited GWAS sample data in EAS ancestry, our analysis might not have had sufficient power to detect more subtle causal relationships. "

**Reviewer #2:**

1.“Univariate” should be corrected as “univariable”

Response: Thank you for pointing out the terminology correction regarding 'univariate' versus 'univariable.' We have made the necessary revisions throughout the manuscript to ensure the correct term 'univariable' is consistently used. We appreciate your attention to detail, which has helped improve the accuracy of our study's presentation.

2. The genetic region for linkage disequilibrium clumping in drug-target MR was unclear.

Response: Thank you for your query regarding the clumping window used for the target gene SNP clumping in our analysis. I appreciate your attention to detail and acknowledge the lack of clarity in our initial description. We have revised the manuscript to accurately reflect the methodology employed.

Revised: "To maximize the instrumental strength of each drug, SNPs utilized as instruments were allowed to exhibit low LD with each other (r2 < 0.30)". Added "localized within ±100 kb windows from gene region" after the paragraph.

3. There was a positive association between TG and endometriosis, but drug-target MR analysis found a negative relation between ANGPTL3-mediated TG and endometriosis. How to explain the results?

Response: Thank you for your inquiry regarding the contrasting results between the positive association of TG with endometriosis and the negative relation observed in drug-target MR analysis involving ANGPTL3-mediated TG. I would like to direct your attention to the discussion section of our manuscript, specifically the penultimate paragraph, where we have addressed this point in detail. In this section, we have explained the potential roles of ANGPTL3 beyond lipid metabolism and its implications for EMS risk. We discussed how activation of ANGPTL3, typically targeted by inhibitors in hypertriglyceridemia treatment, might paradoxically be associated with a reduced risk of EMS. This explanation includes references to studies suggesting ANGPTL3's influence on hormone levels, immune responses, inflammatory processes, and cellular activities relevant to endometriosis. We believe that this discussion provides a comprehensive explanation of the seemingly contradictory findings and highlights the need for further research to unravel the complex mechanisms by which ANGPTL3 and triglyceride levels might influence EMS risk. We appreciate your careful review of our manuscript and hope that this clarification addresses your query effectively.

4. There are some publicly available datasets of eQTLs or pQTLs, did the author consider assessing the findings by exploring the association between ANGPTL3 expression and the outcome, as well as the potential mediators?

Response: Thank you for your suggestion to consider assessing our findings using publicly available datasets of expression quantitative trait loci (eQTLs) or protein quantitative trait loci (pQTLs), particularly in relation to ANGPTL3 expression, the outcome, and potential mediators. Given that our DTMR results presented unexpected findings that are contrary to conventional understanding, we chose not to extend our analysis to include mediation analysis through eQTLs or pQTLs at this stage. Future research could certainly benefit from incorporating these analyses to validate and expand upon our DTMR results. We believe that such studies would be instrumental in elucidating the complex biological mechanisms underlying the associations we have observed and in clarifying the role of potential mediators. We appreciate your insightful recommendation and agree that it points towards an important direction for future research."

5. In line#230, in colocalization analysis, was the 1000 Genomes v3 dataset of European ancestry used as the LD reference panel for analyses of both EUR and EAS ancestry?

Response: Thank you for your question regarding the linkage disequilibrium (LD) reference panel used in the colocalization analysis mentioned in line 230. Similar to the approach detailed earlier in line 140, we utilized the 1000 Genomes Phase 3 reference panel for our LD calculations in both European (EUR) and East Asian (EAS) ancestry analyses. The TwoSampleMR package, which we employed for our MR analysis, incorporates this comprehensive reference panel that includes multiple super-populations, covering a broad spectrum of genetic diversity, including East Asian populations.

6. There are some typos, please go through the manuscript and related materials.

Response: Thank you for bringing to our attention the need for a thorough review of our manuscript and related materials for typographical errors. We appreciate your vigilance in maintaining the quality of the publication. We have carefully gone through the entire manuscript and supplementary materials to correct any typos and ensure the accuracy and clarity of our text. We believe these revisions will enhance the overall readability and precision of our study. We are grateful for your feedback, which has helped improve the quality of our work."

Reference

1.	Yang F, Hu T, Cui H. Serum urate and heart failure: a bidirectional Mendelian randomization study. European Journal of Preventive Cardiology. 2022;29: 1570–1578. doi:10.1093/eurjpc/zwac100

2.	Zhang Y, Tang Z, Tong L, Wang Y, Li L. Serum uric acid and risk of diabetic neuropathy: a genetic correlation and mendelian randomization study. Frontiers in Endocrinology. 2023;14. Available: https://www.frontiersin.org/articles/10.3389/fendo.2023.1277984

3.	Zhang Y, Ni Y, Li L. Genetic Insights into the causal relationship between cannabis use and diabetic phenotypes: A genetic correlation and Mendelian randomization study. Drug Alcohol Depend. 2024;254: 111037. doi:10.1016/j.drugalcdep.2023.111037

4.	Zhang Y, Ni Y, Sun J, Zhao L, Wu B, Ye J. The effect of antioxidant dietary supplements and diet-derived circulating antioxidants on vitiligo outcome: evidence from genetic association and comprehensive Mendelian randomization. Frontiers in Nutrition. 2024;10: 1280162. doi:10.3389/fnut.2023.1280162

5.	Burgess S, Thompson SG. Multivariable Mendelian randomization: the use of pleiotropic genetic variants to estimate causal effects. Am J Epidemiol. 2015;181: 251–260. doi:10.1093/aje/kwu283
