# Supplementary material for: Genetic association of serum lipids and lipid-modifying targets with endometriosis: Trans-ethnic Mendelian-randomization and mediation analysis
Source: PLoS One. 2024 May 31;19(5):e0301752. doi: 10.1371/journal.pone.0301752 (PMC11142702; doi:10.1371/journal.pone.0301752)
Supplement: Attachment — Submitted filename: II-Response to Reviewers.docx [file pone.0301752.s010.docx]

Reviewer #1: The authors of the manuscript “Genetic association of serum lipids and lipid-modifying targets with endometriosis: trans-ethnic Mendelian randomization and Mediation Analysis” have appropriately addressed some of my comments. However, many comments were not adequately addressed. For some comments the authors claim to have changed the manuscript, providing the text change in quotation in the reviewer response, however the given change could not be identified in the “clean” version. Further, for some comments the authors answered the question in the reviewer response but made no attempt at updating the manuscript.


1. The “clean” and “tracked changes” copy of the manuscript are different. The “track changes” manuscript has a different title, “Associations between artificial sweetener intake from cereals, coffee, and Tea and the risk of type 2 diabetes mellitus: a genetic correlation, mediation and mendelian randomization analysis”. As such, my comments refer to the “clean” version only.

Response: I sincerely apologize for the confusion caused by the inclusion of a different manuscript's revisions in the document submitted. This mistake was due to my unfamiliarity with the operating system. I regret any inconvenience this may have caused you during the review process. I have now carefully checked and ensured that the correct version of the manuscript has been submitted without any discrepancies. Thank you for your understanding and patience.

2. No changes to the manuscript text have been made in response to the query concerning the LD reference panel used for variant selection in MR. Please edit the text to detail which 1000 Genomes population was used.

Response: I apologize for the oversight in my previous response to your query about the LD reference panel used for variant selection in our Mendelian Randomization analysis. My misunderstanding led me to believe it was a standalone inquiry, and as a result, I did not amend the manuscript accordingly. I have now corrected this mistake and updated the manuscript to provide a clearer description of the specific 1000 Genomes populations used in our analysis. Thank you for bringing this to my attention, and I appreciate your patience and guidance. See **lines 138-143** for details

Revised:

**Replace** "(ii) The chosen SNPs underwent comprehensive analysis to exclude any associations with potential confounders, ensuring their uniqueness and thereby reducing biases linked to linkage disequilibrium (LD), with strict LD parameters set (r2 < 0.001, clumping distance = 10,000 kb)."

**Revised to** (ii) The chosen SNPs underwent comprehensive analysis, utilizing the East Asian and European populations from the 1000 Genomes Phase 3 reference panel, to exclude associations with the 1000 Genomes Phase 3 reference panel. The selected SNPs underwent comprehensive analysis, utilizing the East Asian and European populations from the 1000 Genomes Phase 3 reference panel, to exclude associations with potential confounders. By setting strict LD parameters (r2 < 0.001, clumping distance = 10,000 kb), we ensured the uniqueness of our SNP selection and minimized LD biases, catering to the genetic diversity of these populations.

3. Regarding the citation of the F statistic formula (line 144), the paper cited does not contain the formula. However, a paper that that paper cites (Burgess et al., 2011) does provide a formula F = ((n-k-1)/k)(R2/(1-R2)). Please cite the original publication. Further, Fan et al., simplifies this formula for k=1, yet claims they use this formula to find an all-SNP F statistic (i.e., “where R2 is the proportion of variance in the exposure explained by the SNPs”). In the following sentence, they make an alternate claim (“we excluded any SNPs with an F-statistic below 10), suggesting they used the formula for one SNP at a time. Please be consistent in your explanation (i.e. from your comments it appears you calculated a SNP-specific F statistic, please update the sentence “where R2 is the proportion of variance in the exposure explained by the SNPs” to “where R2 is the proportion of variance in the exposure explained by the SNP”. Also, please explain how you calculated R2.

Response: Thank you for your valuable feedback. We have carefully revised our manuscript to address your concerns regarding the citation of the F statistic formula and the consistency in our explanation of calculating SNP-specific variance explained (R^2). We have now correctly cited the original publication by Burgess et al., 2011, for the F statistic formula and clarified our approach to calculating the SNP-specific F statistic, ensuring our methodology is accurately represented and consistent throughout the text. We believe these revisions address your comments and enhance the manuscript's clarity and accuracy.

4. You claim to have added “Output from UK Biobank - stratified by sex” – however I cannot find this in text.

Response: I appreciate your attention to detail and realize my response to your earlier query may have been overly simplistic. While I believed that referencing the specific IEU IDs in Table 1 would suffice to address your concerns, I understand now that this approach may not have clearly conveyed the information regarding the "Output from UK Biobank - stratified by sex". Acknowledging this oversight, I have now made further amendments to the manuscript to explicitly include this information within the text, ensuring clarity on the sex-specific nature of the testosterone, estradiol (E2), and sex hormone-binding globulin (SHBG) data sourced from the UK Biobank (UKB). Thank you for guiding me to make these necessary clarifications. See **lines 194-197** for details

Revised:

**Replace** " Additionally, total testosterone (TT), sex hormone-binding globulin (SHBG), and oestradiol (E2) were all obtained from the UK Biobank (UKB)". "

**Revised to** Additionally, total testosterone (TT), sex hormone-binding globulin (SHBG), and oestradiol (E2) datasets, stratified by sex to ensure specificity to female participants, were all obtained from the UK Biobank (UKB).

5. You have not ammended the manuscript text in response to my comment on the two types of horizontal pleiotropy. Additionally, the CAUSE paper indicates the two types of horizontal pleiotropy are (1) correlated pleiotropy and (2) uncorrelated pleiotropy, and that Egger regression and MR-PRESSO both address uncorrelated pleiotropy (Morrison et al., 2020). In line 247 you mention correlated and uncorrelated horizontal pleiotropy. Please explain what these are. Further, if the two types of horizontal pleiotropy are the correlated and uncorrelated horizontal pleiotropy, the sentences containing the phrases (line 246-247) “The CAUSE methodology effectively manages both correlated and uncorrelated horizontal pleiotropy” and (line 250-251) “CAUSE analysis addresses both types of horizontal pleiotropy,” are repetitive and thus could be combined into the one sentence.

Response: Thank you for your valuable feedback regarding the description of horizontal pleiotropy within our manuscript. Initially, I misunderstood your request as a simple inquiry for clarification, and therefore, did not amend the text to include a detailed explanation. I appreciate your guidance in highlighting this oversight. Following your advice, I have revised the manuscript to eliminate the repetitive statements and have now included a succinct yet comprehensive description of both correlated and uncorrelated horizontal pleiotropy, as distinguished in the CAUSE methodology per Morrison et al. (2020). This adjustment ensures a clearer understanding of the terms and reinforces the robustness of our analytical approach. See **lines 253-263** for details

Revised:

**Replace** " When unavoidable horizontal pleiotropy was detected by MR-PRESSO, the causal analysis using summary effect estimates (CAUSE) was applied[37]. The CAUSE methodology effectively manages both correlated and uncorrelated horizontal pleiotropy within genome-wide datasets. This approach utilizes genome-wide summary statistics to determine whether variant effects align with expected causal impacts. Unlike traditional MR methods, which rely on rigid assumptions, CAUSE analysis addresses both types of horizontal pleiotropy, thereby reducing the risk of false-positive results[37]. "

**Revised to** When unavoidable horizontal pleiotropy was detected by MR-PRESSO , the causal analysis using summary effect estimates (CAUSE) methodology was applied[37], effectively managing both correlated and uncorrelated horizontal pleiotropy within genome-wide datasets[37]. Correlated horizontal pleiotropy, wherein genetic variants influence multiple traits through shared heritable factors, presents a particular challenge by potentially mimicking causal relationships. CAUSE differentiates these effects from true causal relationships by utilizing genome-wide summary statistics, thus addressing both types of horizontal pleiotropy and significantly reducing the risk of false-positive results. This approach contrasts with Egger regression and MR-PRESSO, which primarily focus on uncorrelated horizontal pleiotropy by adjusting for pleiotropic effects that are independent of the genetic variant's association with the exposure. CAUSE thereby provides a comprehensive framework for analysis that surpasses traditional MR methods in flexibility and robustness.

6. Line 313 “Supplementary methods also yielded consistent findings.” – you have not addressed my question about what the supplementary methods are, nor amended the text based on this comment about what the supplementary methods are. Please explain what the particular methods are, and please include the reference to S3 table at the end of the sentence. For example: “Supplementary methods MR Egger, weighted median and CML also yielded consistent findings (S3 Table)”

Response: My sincerest apologies for the oversight in my previous responses regarding the supplementary methods used in our analysis. Initially, I did not fully grasp the specificity of your request. I have now corrected this misunderstanding and amended the manuscript accordingly to explicitly detail the supplementary methods employed—MR Egger, weighted median, and CML—and have included a reference to S3 Table at the end of the sentence to ensure clarity and comprehensiveness of the information provided. See **lines 322-323** for details

Revised:

**Replace** " Supplementary methods also yielded consistent findings "

**Revised to** Supplementary methods MR Egger, weighted median and CML also yielded consistent findings (S3 Table)

7. The updated title for table 2 detailed in the response to reviewers has not been implemented in text (line 347). Further, the MVMR results for all tested exposures should be available in the manuscript. Whilst I appreciate adding those results to the table will increase the length and thus the ease of reading, reporting all results is important for clarity and transparency. If you are concerned about Table 2 being too long, other solutions are possible, such as including the full set of results as a supplementary table instead of in text.

Response: Thank you for your continued guidance and constructive suggestions. Upon reflecting on your initial advice, I have now followed through by incorporating estimates for both exposures in the models, as you originally recommended. This adjustment has been made to ensure clarity and transparency in our findings, directly addressing your concerns. The revised **Table 2**, which now includes the estimates of both exposures within the models, has been updated accordingly in the manuscript. I appreciate your patience and understanding as we strive to improve the clarity and comprehensiveness of our work.

8. The response to the query about the CAUSE result (Query 10 within results) has not been implemented in text (line 334-335).

Response: I deeply apologize for the oversight in my previous submission, which led to the omission of the response to your query about the CAUSE result in the text you reviewed. The mistake occurred due to my error in not providing the correctly marked manuscript for your evaluation. I have now rectified this issue, and in the revised, marked manuscript, the response has been highlighted for clarity, specifically between **lines 338-340**. I regret any confusion this may have caused and appreciate your understanding and patience as we ensure all your concerns are addressed comprehensively.

9. Line 201 – the weighted median method is consistent when up to 50% of the information comes from invalid instrumental variables. Here you have indicated it is valid if >50 % of the information comes from invalid instrumental variables (through use of the term “a majority of the genetic variants”), which is not true. Please rephrase this sentence.
Response: Thank you for your critical observation regarding the description of the weighted median method's robustness in line 201 of our manuscript. I have now revised this sentence to accurately reflect that the weighted median method remains robust when up to 50% of the information comes from potentially invalid instrumental variables, providing a more precise description of its utility. I appreciate your guidance in ensuring the accuracy of our methodological descriptions. See **lines 207-210** for details

Revised:

**Replace** "The weighted median method becomes pivotal when a majority of the genetic variants are considered invalid"

**Revised to** The weighted median method remains robust when up to 50% of the information is derived from potentially invalid instrumental variables, allowing for reliable estimation

10. The manuscript would be further enhanced by explaining the difference between a mediator and a confounder.
Response: Thank you for your constructive feedback regarding the differentiation between mediators and confounders in our analysis. We have addressed this by clearly defining and distinguishing between these two critical concepts directly within the text. This clarification is now included following the initial discussion of our mediation and confounding factor findings, enhancing the manuscript's clarity and aiding in the accurate interpretation of our results. Specifically, we have elucidated the roles of diastolic blood pressure (DBP), systolic blood pressure (SBP), and triglycerides (TG) in the context of our study's findings, providing a clearer understanding of their respective roles as confounders and mediators in the relationship between serum lipids and endometriosis. We believe this addition satisfactorily addresses your concern and enhances the manuscript's contribution to the field. See **lines 351-356** for details
